# Targeting Biliary Tract Cancers with Antibody–Drug Conjugates: Advances in Molecular Targets and Rational Combinations

**DOI:** 10.3390/cancers18040600

**Published:** 2026-02-12

**Authors:** Yulin Chen, Chidan Wan, Chen Shi, Xiong Cai

**Affiliations:** 1 Department of Hepatobiliary Surgery, Union Hospital, Tongji Medical College, Huazhong University of Science and Technology, Wuhan 430022, China; 2024xh8007@hust.edu.cn (Y.C.); wanchidan@hust.edu.cn (C.W.); 2 Department of Pharmacy, Union Hospital, Tongji Medical College, Huazhong University of Science and Technology, Wuhan 430022, China; 3 Hubei Province Clinical Research Center for Precision Medicine for Critical Illness, Wuhan 430022, China

**Keywords:** biliary tract cancers, cholangiocarcinoma, antibody–drug conjugates, HER-2, immunotherapy combinations

## Abstract

Antibody–drug conjugates (ADCs), an emerging targeted treatment method, are designed to couple the tumor selectivity of monoclonal antibodies with the cytotoxic potency of small-molecule payloads, thereby offering an accurate approach for biliary tract cancers (BTCs). BTC is characterized by typical molecular heterogeneity, a desmoplastic stroma, and an immune-suppressive microenvironment, which together limit the efficacy of conventional chemotherapy and weaken responses to immunotherapy in unselected patients. The demand for drugs that can improve the survival rate of BTC patients has not yet been met. The evaluation of ADC therapy for BTC has become a focus of extensive clinical and preclinical research, and encouraging preliminary results have been achieved. In this review, we comprehensively summarize the increasing body of evidence regarding the application of ADCs in BTC relative trials. Starting with a brief discussion of the functional principles of ADCs, we summarize the trials of different designs of ADCs in BTC, as well as the relevant clinical data related to ADC-based combination therapy regimens.

## Simple Summary

Biliary tract cancers are difficult to treat because they are often diagnosed late and because many drugs cannot effectively reach or control the tumor. Antibody–drug conjugates are a new type of targeted medicine that links an antibody, which can recognize a marker on cancer cells, to a highly potent anti-cancer drug. This design aims to deliver the drug more directly to tumor cells and reduce damage to normal tissues. In this review, we summarize which tumor markers are being tested for these medicines in biliary tract cancers, what clinical studies have shown so far, and the explorations related to the combination of antibody–drug conjugates with other treatment methods (such as immunotherapy or targeted drugs). Our goal is to provide an updated and practical overview that helps researchers and clinicians understand current progress, identify promising directions, and guide future study design in this rapidly developing field.

## 1. Introduction

Biliary tract cancers (BTCs) are a heterogeneous group of malignancies arising from the gallbladder, cystic duct, and biliary tree. Although clinical trials often group them together, BTC is not a single disease. BTC encompasses gallbladder cancer (GBC) and cholangiocarcinoma (CCA), the latter of which is classified by anatomical site into intrahepatic (iCCA), perihilar (pCCA), and distal (dCCA) subtypes [1]. These distinctions are clinically significant, as each subtype possesses a unique epidemiological, molecular, and biological profile [1,2]. While historically considered rare, the global incidence of BTC is rising. Recent data indicate that CCA alone accounts for roughly 15% of primary liver cancers and 3% of gastrointestinal malignancies, reflecting a significant and growing public health burden [2].

However, the clinical management of BTC remains challenging largely due to intrinsic biological barriers that limit therapeutic efficacy. Histologically, BTCs exhibit a prominent desmoplastic reaction in which malignant glands are embedded within dense stroma populated by fibrogenic and immune cell subsets. This fibrotic matrix not only fosters spatial heterogeneity but also creates a significant barrier to drug penetration, often rendering conventional systemic therapies ineffective [3]. In addition, these tumors are typically immunologically ‘cold’ and possess a suppressive microenvironment that dampens the response to standard immune checkpoint inhibitors (ICIs). Clinically, the frequent diagnosis of BTC at an advanced stage renders systemic therapy the mainstay of treatment [2]. For over a decade, gemcitabine plus cisplatin (GemCis) served as the standard of care [4], a benchmark recently evolved by the addition of ICIs (durvalumab or pembrolizumab). While phase III trials such as TOPAZ-1 and KEYNOTE-966 confirm that the addition of immunotherapy improves survival, the survival benefit remains incremental because the immunosuppressive and fibrotic microenvironment described above constrains efficacy [5,6].

Over the past two years, the conventional paradigm of chemo-immunotherapy has yielded to a more personalized approach rooted in precision oncology. In this model, the deployment of targeted therapies is driven by the distinct molecular profile of an individual’s tumor, ensuring that therapeutic intervention is precisely matched to the driver mutations identified through genomic profiling. Current National Comprehensive Cancer Network (NCCN) Guidelines (Version 2.2025) have incorporated several targeted agents into the therapeutic framework for BTC [7]. However, given the rapid pace of clinical trial readouts, these recommendations represent an evolving standard subject to ongoing refinement. These recommendations include inhibitors targeting IDH1 mutations (e.g., ivosidenib), FGFR2 fusions or rearrangements (e.g., pemigatinib, futibatinib), and human epidermal growth factor receptor 2 (HER2, also known as ERBB2) amplification/overexpression (e.g., trastuzumab deruxtecan, T-DXd). Furthermore, tissue-agnostic approvals for tumors harboring NTRK fusions, RET fusions, or microsatellite instability–high (MSI-H) status have further expanded the precision therapeutic options [7].

The evolution of targeted therapy has focused on achieving selective tumor eradication while sparing normal tissues. While small-molecule inhibitors target intracellular signaling, monoclonal antibodies (mAbs) have served as the primary tools for targeting extracellular antigens and surface receptors. Despite their potential, mAbs as monotherapies face significant hurdles in complex solid tumors like BTCs. Challenges such as uneven antigen expression, physical barriers from the desmoplastic stroma, and compensatory signaling often curtail their efficacy. Antibody–drug conjugates (ADCs) address these limitations by acting as targeted delivery vehicles. ADCs deliver a cytotoxic payload directly to the tumor, where internalization and intracellular release ensure potent cell killing with reduced systemic toxicity [8]. This strategy is particularly salient in BTCs, where it may effectively bypass the malignancy’s intrinsic biological barriers [2,3].

This review provides a comprehensive synthesis of the clinical landscape of ADCs in BTC, integrating evidence from the peer-reviewed literature and recent conference updates. The clinical landscape of BTC is currently undergoing a shift from conventional chemotherapy to precision medicine. While large-scale Phase III data is still maturing, the rapid proliferation of early-phase trials has created a complex array of targetable options. A timely synthesis of these emerging agents is critical not only for guiding patient enrollment into appropriate clinical trials but also for informing the design of next-generation combination strategies. We focus on subtype-specific outcomes across iCCA, pCCA, and dCCA, as well as gallbladder cancer, outlining the structural and biological determinants of efficacy. Furthermore, we explore rational combination strategies designed to overcome resistance and modulate the tumor microenvironment. Finally, we highlight key challenges and prospective directions to clarify the mechanisms underlying differential responses to ADCs in this heterogeneous disease.

## 2. Rationale and Mechanism

### 2.1. Mechanism of ADCs

ADCs are modular therapeutics that link a tumor-targeting monoclonal antibody to a potent cytotoxic payload via a chemical linker. In the classical mechanism of action, the ADC binds to a cell-surface antigen, is internalized via endocytosis, and trafficked to lysosomes, where chemical or enzymatic processing enables payload release and tumor cell killing [9]. Recently, the therapeutic landscape has expanded with the development of non-internalizing ADCs. Unlike classical ADCs that require receptor-mediated endocytosis, these agents utilize linkers cleavable by extracellular proteases or chemical triggers within the tumor microenvironment (TME). This mechanism allows the payload to be released extracellularly and diffuse into tumor cells, thereby targeting antigens with poor internalization rates or those expressed on the stromal matrix. A notable example involves ADCs targeting the tumor sub-endothelium or extracellular matrix components. However, this approach involves the risk of off-target release in healthy tissues if the triggering conditions (e.g., specific protease activity) are not strictly confined to the tumor, potentially increasing systemic toxicity [10,11]. Consequently, this advancement allows for the targeting of matrix antigens and antigens with poor internalization rates, significantly broadening the potential scope of ADC application.

**Figure 1:**
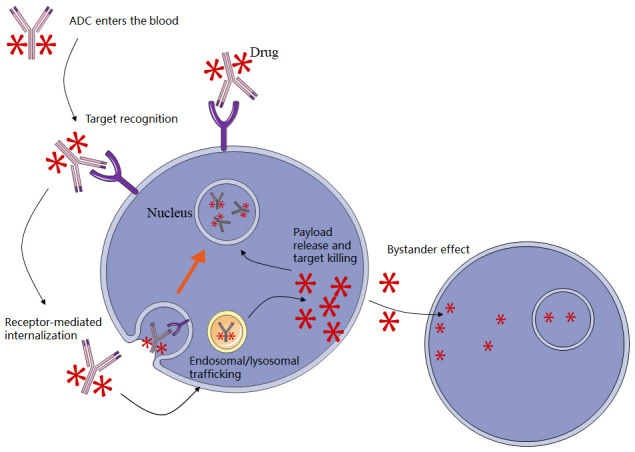
Schematic workflow of ADC action in BTC. After binding to a surface antigen, the ADC-antigen complex undergoes receptor-mediated internalization and trafficking to endosomes/lysosomes. The linker is cleaved (or the antibody is degraded) to release the cytotoxic payload, which induces tumor cell death through DNA damage or mitotic arrest. Membrane-permeable payloads may generate a bystander effect and kill adjacent antigen-low/negative cells, which is relevant for intratumoral heterogeneity in BTC.

The cytotoxic efficacy of ADCs is dictated by the payload. Standard payloads primarily function by disrupting microtubules (e.g., auristatins, maytansinoids) or inducing DNA damage (e.g., topoisomerase I inhibitors). Importantly, the payload classes dictate the distinct toxicity profile of the ADC. Microtubule inhibitors, such as monomethyl auristatin E (MMAE), are frequently associated with cumulative peripheral neuropathy and neutropenia. In contrast, topoisomerase I inhibitors (e.g., DXd, SN-38) exhibit a different safety spectrum; while generally causing less neurotoxicity, they carry a specific risk of interstitial lung disease (ILD) or pneumonitis, necessitating vigilant respiratory monitoring. Recently, payload categories have expanded to include proteasome inhibitors and anti-apoptotic protein inhibitors [12]. Furthermore, to mitigate drug resistance, ‘dual-payload’ ADCs have been developed to deliver discrete payloads in a complementary or synergistic mode [13]. Membrane permeability is a critical feature of payload selection, enabling hydrophobic payloads released from the antibody to diffuse out of the target cell into neighboring cells. This phenomenon, known as the “bystander effect,” allows for the killing of surrounding cells (Figure 1) [9,14].

Evolution of ADCs:

• First-generation ADCs (e.g., gemtuzumab ozogamicin) relied on murine antibodies and acid-labile linkers. However, their clinical utility was frequently compromised by high immunogenicity and instability, leading to premature payload release in circulation.
• Second-generation ADCs (e.g., ado-trastuzumab emtansine, T-DM1) addressed these safety concerns by incorporating humanized monoclonal antibodies and stable, non-cleavable linkers. While this reduced systemic toxicity, these agents were limited by stochastic conjugation methods that yielded heterogeneous drug-to-antibody ratios (DARs), as well as the use of charged payloads that lacked the ability to induce a bystander effect.
• Third-generation ADCs (e.g., trastuzumab deruxtecan, T-DXd) overcome these limitations through advanced conjugation technologies and the use of cleavable peptide linkers. These innovations enable a consistently high DAR and the release of membrane-permeable payloads, thereby successfully generating the bystander effect to target surrounding tumor clones.

### 2.2. Circumventing Pathophysiological Barriers of BTC

The ‘bystander effect’ represents a theoretical advantage for ADCs in the treatment of BTC, aiming to counter the malignancy’s intrinsic heterogeneity. By releasing membrane-permeable cytotoxic payloads, ADCs may extend their therapeutic reach beyond the primary target cell to neighboring antigen-negative clones. This mechanism is particularly relevant for overcoming the spatial heterogeneity often observed in BTC. Nevertheless, the dense desmoplastic stroma remains a significant physiological hurdle. While the bystander effect improves the probability of eliminating tumor cells shielded by fibrosis compared to non-cleavable linkers, the extent of drug penetration varies and remains dependent on the specific physicochemical properties of the payload and the localized tumor architecture [12,15].

Unlike conventional targeted therapies that are limited to cells explicitly expressing a target antigen, these permeable payloads exert a potent field effect. Once released intracellularly, the payload diffuses across the membrane of the primary target cell into the surrounding microenvironment, eradicating neighboring antigen-negative clones and supportive stromal cells that would otherwise survive monospecific blockade. By effectively saturating the local tumor milieu, this bystander effect serves as a critical countermeasure to the intratumoral heterogeneity and fibrotic barrier that drive therapeutic resistance in BTC [15,16].

Furthermore, ADCs are uniquely positioned to reverse the ‘cold’ immune phenotype characteristic of BTC. The cytotoxic payloads delivered by these agents, which are amplified by the bystander killing of surrounding tissue, frequently induce immunogenic cell death (ICD) [17]. This regulated cell death process releases damage-associated molecular patterns (DAMPs) such as calreticulin, HMGB1, and ATP. These danger signals act as potent immunological adjuvants that recruit dendritic cells and promote the infiltration of effector T-cells into the previously excluded tumor core [17,18]. Consequently, this mechanism facilitates the transformation of the tumor from a ‘cold’ to a ‘hot’ state. Such remodeling of the tumor microenvironment amplifies the antitumor immunity and provides a robust biological rationale for combinatorial strategies with ICIs.

## 3. ADC Targets in BTC

### 3.1. Target Selection

The selection of ADC targets fundamentally aims to maximize the therapeutic window, deliver the payload to the tumor, and limit extratumoral binding. Generally speaking, the optimal antigen should be (i) extracellular/cell surface (therefore circulating ADC can bind to it), with the antigen abundance being as high as possible, because the number of ADC reaching tumor cells is positively correlated with the antigen abundance [19,20]; (ii) targeting tumor-associated antigens rather than those expressed in normal tissues, thereby reducing the toxicity to normal tissues [19]; (iii) non-secretory (to avoid “antigen aggregation” binding in the circulation); and (iv) effectively internalizing after antibody binding to ensure the release of the effective payload within the cells [9]. Additionally, in solid tumors, further requirements are that the antigen has sufficient and relatively uniform expression on the tumor surface, because uneven or low-density expression may reduce the effective payload delivery throughout the lesion. Even though theoretically ADC can kill these tumor cells through bystander effects, some studies have shown that even though it can demonstrate effective killing based on bystander effects in preclinical studies, it may not necessarily reflect clinically meaningful activity in actual clinical trials [21,22].

### 3.2. Clinically Validated Targets

#### 3.2.1. ERBB-Family

• HER2 (ERBB2)

The ERBB receptor family remains the most clinically mature antigen class for antibody–drug conjugates (ADCs) in biliary tract cancers (BTC), largely because actionable HER2 alterations are repeatedly observed across BTC subtypes and can be therapeutically exploited [23]. As a member of the HER RTK family, HER2 possesses strong catalytic kinase activity, which is triggered upon dimerization [24]. The HER2/HER3 complex, in particular, acts as a potent driver of the oncogenic PI3K/AKT signaling axis [25]. Epidemiologically, HER2 overexpression is distinctively enriched in extrahepatic cholangiocarcinoma (eCCA) compared to iCCA, with reported prevalence rates of approximately 14–20% versus 5–6%, respectively [26]. Despite its biological significance, HER2 status has not been shown to correlate with poor prognosis or reduced sensitivity to chemotherapy. In contemporary profiling work, HER2 expression is estimated in roughly a minority subset of BTC and appears more frequent in extrahepatic than intrahepatic disease, supporting HER2 as a rational ADC target rather than a universal marker. Importantly, T-DXd has granted U.S. FDA accelerated approval in a tumor-agnostic setting for previously treated, unresectable or metastatic HER2-positive (IHC 3+) solid tumors. While this creates a practical regulatory framework for access, it is important to note that full approval remains contingent upon verification of clinical benefit in confirmatory trials.

T-DM1 represents an early attempt to target HER2 in CCA using an ADC platform. Structurally, it conjugates trastuzumab to the payload DM1 via a stable, non-reducible thioether linker. While effective in preclinical models, where it successfully induced regression in HER2-positive xenografts via mechanisms involving cell cycle arrest and blockade of HER2-HER3 signaling, the clinical results have been disappointing. In the KAMELEON phase II study (NCT02999672), recruitment challenges led to premature termination, and the drug demonstrated limited activity, with only one partial response recorded among seven evaluable patients with biliary or pancreatic malignancies [27,28]. The disconnect between preclinical efficacy and clinical outcomes is likely multifactorial. While the non-cleavable linker prevents the bystander killing effect needed to address tumor heterogeneity, other contributing barriers include the dense desmoplastic stroma that limits macromolecular penetration and potential intrinsic resistance to microtubule-inhibitor payloads in refractory BTC populations.

T-DXd represents a significant structural evolution in ADC design. It comprises a humanized anti-HER2 antibody conjugated to a topoisomerase I inhibitor payload (DXd) via a cleavable tetrapeptide linker, achieving a high drug-to-antibody ratio (DAR) of approximately 8. This configuration grants the drug exceptional systemic stability, and it’s characterized by a low payload shedding rate of 1.2–3.9%, while ensuring efficient cellular internalization [29]. Mechanistically, T-DXd functions as a dual-action therapeutic: it retains the inherent biological activities of trastuzumab, such as ADCC induction and pAKT signaling suppression, while delivering a potent cytotoxic payload that triggers DNA damage and subsequent apoptosis [29]. Following its regulatory approval for breast, gastric, and lung cancers, T-DXd has been investigated in biliary tract cancers (BTCs). The Phase II HERB trial enrolled 32 patients, stratified by HER2 status. In the HER2-positive cohort, T-DXd monotherapy elicited an objective response rate (ORR) of 36.4% (including two complete responses), with a median progression-free survival (mPFS) of 4.4 months and median overall survival (mOS) of 7.1 months. Notably, activity was also observed in HER2-low patients, who achieved an ORR of 12.5% and an mOS of 8.9 months (Table 1) [30,31,32,33,34,35,36,37,38,39,40,41,42]. However, safety remains a consideration, as Grade ≥ 3 treatment-related adverse events (TRAEs) were reported in 81.3% of participants, resulting in a discontinuation rate of 25%. Further validation came from the DESTINY-PanTumor02 Phase II trial (NCT04482309), which evaluated T-DXd in pre-treated HER2-expressing solid tumors. In the primary analysis (median follow-up 12.75 months), the overall cohort (*n* = 267) demonstrated an ORR of 37.1% and a robust mOS of 21.1 months. Specifically within the BTC sub-cohort, the ORR was 22%, with Grade ≥ 3 adverse events recorded in 40.8% of the total population [43].

GQ1001 represents a next-generation evolution of the trastuzumab-DM1 format, utilizing a novel ‘intelligent ligase-dependent conjugation’ (iLDC) technology to achieve site-specific linkage. This design significantly enhances linker stability. Notably, previous in vivo assays revealed a payload shedding rate of only 1% compared to T-DM1, translating to reduced off-target toxicity in PDX models while maintaining comparable antitumor potency. Based on these superior preclinical data, a Phase I trial (NCT04450732) is currently investigating GQ1001 in patients with HER2-positive advanced solid tumors, including those with biliary tract cancer [44].

Disitamab vedotin (DV, Aidexi^®^) represents a novel ADC format comprising hertuzumab, which is a humanized anti-HER2 antibody with superior affinity compared to trastuzumab, conjugated to the microtubule disruptor MMAE via a cleavable mc-val-cit-PABC linker (DAR ≈ 4). This design confers distinct biological advantages: hertuzumab elicits more potent antibody-dependent cellular cytotoxicity (ADCC), while the cleavable linker facilitates a strong bystander killing effect [45]. Consequently, unlike T-DM1, DV maintains efficacy even in tumors with heterogeneous or lower HER2 expression (e.g., IHC 2+/FISH−), a finding validated by preclinical superiority over naked antibodies or free toxins [46].

MRG002 represents a distinct approach in ADC design, comprising the humanized anti-HER2 antibody MAB802 conjugated to MMAE via a cleavable valine–citrulline linker (average DAR ~3.8) [47]. Although MAB802 shares the amino acid sequence of trastuzumab, it is distinguished by extensive core fucosylation of its Fc region. This glyco-engineering modification intentionally restricts binding to Fcγ receptors, thereby significantly attenuating ADCC [48]. Despite this reduced immune engagement, MRG002 exhibits efficient internalization and potent cytotoxicity. Preclinical comparisons demonstrated that MRG002 possesses superior antitumor activity relative to T-DM1 in both HER2-high and HER2-low patient-derived xenograft (PDX) models, supported by a favorable pharmacokinetic profile [47]. Clinical verification was explored in a Phase I study of HER2-positive solid tumors, where preliminary results showed the agent achieved an objective response rate (ORR) of 43% and a disease control rate (DCR) of 81% in 25 evaluable patients [49]. Currently, the safety and efficacy of MRG002 specifically in advanced BTC are being investigated in a Phase II trial (NCT04837508), with results eagerly anticipated [50].

• HER3 (ERBB3)

HER3 (ERBB3) is a kinase-impaired ERBB-family receptor that primarily functions as a signaling “adaptor” through heterodimerization (most often with HER2 or EGFR, epidermal growth factor receptor), thereby activating downstream survival pathways such as PI3K/AKT. In BTC, HER3 expression appears common and may be dynamic under therapeutic pressure: in an ASCO report analyzing previously treated BTC samples, HER3 IHC positivity (≥1+) was observed in a large majority of cases, and upregulation after chemotherapy was noted in some paired specimens [51]. Although the BTC-specific prognostic value of HER3 remains insufficiently defined in cohorts, earlier data in resected extrahepatic cholangiocarcinoma suggested that HER3 overexpression correlated with worse survival, supporting biological relevance while also highlighting the need for contemporary validation [52]. However, clinical evidence in BTC specifically is not yet mature. As of the latest publicly accessible records, patritumab deruxtecan is being evaluated in a phase 1/2 study for selected gastrointestinal cancers that include BTC (HERTHENA-PanTumor02; NCT06596694), with typical early-phase endpoints such as safety, tolerability and objective response assessment. However, BTC cohort efficacy outcomes have not been clearly reported in peer-reviewed form or in openly accessible conference materials; therefore, any claim of BTC response rates for HER3-DXd at this time should be treated as unavailable rather than inferred from other tumor types [53].

• EGFR (ERBB1)

EGFR is frequently expressed in BTC, but signal-blockade with anti-EGFR monoclonal antibodies has not translated into meaningful survival benefit when added to gemcitabine-based first-line chemotherapy. A 2020 systematic review and meta-analysis of randomized trials found no significant improvement in OS, PFS, or ORR with EGFR mAbs, while grade 3–4 toxicities (notably skin rash and hematologic AEs) increased, supporting the view that EGFR is an unreliable therapeutic dependency in unselected BTC populations [54]. Against this background, EGFR-directed ADCs reposition EGFR primarily as a cell-surface molecular target for payload delivery, rather than as a pathway to be inhibited. MRG003 is one such program; it is described as a fully human anti-EGFR IgG1 conjugated to MMAE via a protease-cleavable valine–citrulline linker, and multiple phase II studies, which included a BTC study, have been registered [55]. The BTC focused trial (NCT04838964) is a single-agent phase II study in unresectable locally advanced or metastatic, centrally confirmed EGFR-positive BTC after at least one prior standard therapy, with staged enrollment (phase IIa safety/preliminary efficacy followed by expansion if criteria are met). As of now, peer-reviewed BTC efficacy outcomes for MRG003 are not readily available, so its effect still needs to be further observed [32].

#### 3.2.2. TROP2

TROP2 is among the most frequently detectable ADC antigens in cholangiocarcinoma tissue series, making it a pragmatic candidate for BTC where broad applicability is otherwise difficult to achieve. In a recent profiling cohort (*n* = 23), TROP2 positivity was reported in 82.6% of tumors (with variable intensity/H-scores), but the study also noted that benign biliary epithelium can stain, raising an on-target/off-tumor consideration that is particularly relevant in an inflamed, obstructed biliary system [23]. Consistently, a larger tissue microarray analysis reported high TROP2 positivity across cholangiocarcinoma samples, but did not find clear associations with survival metrics, supporting TROP2 more as a delivery marker than a prognostic biomarker [56].

Clinically, TROP2 targeting in BTC is being advanced through both disease-dedicated and pan-tumor frameworks. Sacituzumab govitecan has BTC-specific translational support in cholangiocarcinoma organoid models and is being tested in a dedicated phase II trial in previously treated cholangiocarcinoma (SIGNA; NCT06178588). At present, BTC efficacy outcomes remain waiting for peer-reviewed reporting, so the most defensible statement is that this program will provide the first prospective BTC-focused readout for a TROP2-ADC [37,56]. In parallel, datopotamab deruxtecan (Dato-DXd) includes a biliary tract cancer substudy within the TROPION-PanTumor03 master protocol (NCT05489211), and additional TROP2-ADCs (e.g., OBI-902, NCT07124117) are entering early testing with cholangiocarcinoma intent [38,41].

#### 3.2.3. Claudin 18.2

Compared with broadly expressed epithelial antigens, CLDN18.2 is a narrower but potentially higher-specificity target in BTC. In a recent cholangiocarcinoma profiling study (*n* = 23), CLDN18.2 positivity was uncommon (13.0%), indicating that CLDN18.2-directed ADC strategies will likely require biomarker enrichment and concentrated IHC workflows instead of including all participants [23]. Clinically, CLDN18.2-ADC development relevant to BTC currently rests mainly on early-phase and trial-registry evidence. IBI343 is being evaluated in a first-in-human phase Ia/Ib study that includes advanced solid tumors such as pancreatic cancer and BTC (NCT05458219), and an ASCO 2024 abstract reports preliminary safety/efficacy observations in CLDN18.2-positive PDAC and BTC [57]. In parallel, a BTC-dedicated combination study is recruiting CLDN18.2-positive advanced BTC patients for LM-302 (a CLDN18.2-ADC) plus cadonilimab after failure of standard therapy (NCT05994001), which is worth noting because it directly tests whether ADC–immunotherapy pairing can overcome BTC resistance patterns [42].

#### 3.2.4. B7-H4

B7-H4 is an inhibitory B7-family immune checkpoint molecule. In cholangiocarcinoma, increased B7-H4 expression has been linked to immune suppression (reduced cytotoxic T-cell activity) and tumor progression, so it is biologically reasonable to consider B7-H4 as a therapeutic target in BTC [58]. Experimental and translational reports in intrahepatic cholangiocarcinoma also suggest that B7-H4-related pathways can support immune evasion and aggressive behavior, which provides a disease-relevant rationale for targeting this antigen on tumor cells or within the tumor microenvironment [59]. Clinically, the leading B7-H4 ADC program that clearly includes BTC is puxitatug samrotecan (AZD8205), an anti-B7-H4 monoclonal antibody conjugated (via a cleavable linker) to a topoisomerase I inhibitor payload; this agent is being tested in the first-in-human BLUESTAR trial (NCT05123482), which includes a biliary tract cancer cohort [40,60]. Initial dose-escalation results reported in Annals of Oncology described a safety profile broadly consistent with other TOP1i ADCs and showed initial antitumor activity in heavily pretreated solid tumors, but BTC-specific response tables are not clearly available in the public report, so BTC efficacy should be described as not yet confirmed [61]. Other B7-H4 ADCs (e.g., SGN-B7H4V) are also in early clinical development in advanced solid tumors, which supports target feasibility, but again BTC subgroup evidence is limited at present [62].

#### 3.2.5. NECTIN4 (Nectin-4)

NECTIN4 is a promising ADC antigen in BTC because it may offer a better tumor–normal contrast than some epithelial targets. In a recent cholangiocarcinoma profiling cohort, NECTIN4 was positive in 65.2% of tumors, and about one-third showed 2+/3+ intensity; importantly, benign bile ducts were reported as negative, suggesting potential tumor selectivity in BTC [23]. NECTIN4 is already a validated ADC target (enfortumab vedotin is a Nectin-4-targeted ADC), and early-phase NECTIN4-ADC programs have started to include Gallbladder/Biliary disease sites, but BTC-specific efficacy data are still limited and remain in progress [63].

### 3.3. Other Potential Targets

#### 3.3.1. B7-H3

B7-H3 is an immune related surface protein that can be detected in a subset of cholangiocarcinoma specimens, but the staining pattern may include stromal compartments, so tumor selectivity can be an issue for ADC development. In one recent profiling study of resected cholangiocarcinoma samples, B7-H3 positivity was observed in about half of cases, while CLDN18.2 was less frequent, suggesting that B7-H3 could be relevant but may not be a universal target in unselected BTC. Based on this background, a B7-H3 directed DXd ADC, ifinatamab deruxtecan, is being tested in a multi-cohort clinical study that explicitly includes a BTC cohort, which will be important to clarify activity and safety in this disease (Table 2) [23,39].

#### 3.3.2. ICAM1 (CD54)

ICAM1 is a cell adhesion molecule that may fit BTC biology because it is linked with inflammatory signaling and it can mediate efficient internalization, which is a key requirement for ADC payload delivery. A recent preclinical study screened multiple cholangiocarcinoma cell lines for surface antigens and identified ICAM1 as a candidate target, then constructed two ICAM1 ADCs using cleavable linkers with either a DXd derivative payload or MMAE. In vitro and in vivo, both ICAM1 ADCs showed strong antitumor activity and were compared with gemcitabine as a reference, supporting the feasibility of ICAM1 based ADC delivery in cholangiocarcinoma models. At present, this evidence is still preclinical, so the real value in BTC will depend on future confirmation in patient cohorts and clinical trials [64].

#### 3.3.3. Glypican 1 (GPC1)

GPC1 is attractive mainly because it may offer a wider therapeutic window in BTC, and it may also provide additional biology-driven value since it can be present on tumor cells and tumor-associated vessels. In a preclinical report, GPC1 expression was confirmed in cholangiocarcinoma cells and tissues, and immunohistochemistry of extrahepatic cholangiocarcinoma samples showed a substantial subgroup with high GPC1 expression. The authors developed an MMAF conjugated GPC1 ADC and reported tumor growth inhibition in vitro and in vivo, with evidence suggesting both direct tumor cell killing and an antiangiogenic component through effects on tumor vessel endothelium. These findings are promising but still at the preclinical stage, so translation to BTC patients require further assessment [65].

#### 3.3.4. CD44 Variant 5 (CD44v5)

CD44 variant isoforms are sometimes more tumor-enriched than the standard CD44 form, which can be helpful for reducing on-target/off-tumor risk in BTC. A 2023 study reported that CD44v5 was present on the surface of many intrahepatic cholangiocarcinoma tumors and then developed a CD44v5 targeted ADC using an MMAE payload and a cleavable valine citrulline linker. The study also highlighted a practical point for BTC, namely that protease activity such as cathepsin B can support intracellular payload release, and the ADC showed tumor regression in patient derived xenograft models without obvious toxicity signals in the reported experiments. This target is still exploratory for BTC because there is no mature clinical dataset yet, but it represents a new type of biomarker-driven therapeutic option [66].

#### 3.3.5. Tumor-Associated MUC1 (TA-MUC1)

TA-MUC1 is a tumor-associated form of MUC1 with altered localization and glycosylation in cancers, and it is being explored as an ADC target in several epithelial tumors, including BTC. DS-3939a is a TA-MUC1 directed DXd ADC now in a phase 1/2 first in human program. At present, due to the lack of corresponding clinical data, it is impossible to determine its efficacy. However, it remains a promising therapeutic target [67].

Taken together, the current ADC landscape in BTC shows an uneven maturity across targets. HER2 has the most disease-specific clinical evidence and also benefits from a tumor-agnostic regulatory pathway, so it can be discussed as the leading reference for efficacy and key risks. Several other targets, such as EGFR, TROP2, CLDN18.2, B7-H4, and B7-H3, are already being tested in BTC-relative trials, but BTC subgroup results are still limited in many programs. In parallel, exploratory targets supported mainly by preclinical data (for example ICAM1, GPC1, TA-MUC1) provide a reference for future studies, but they need standardized assays and prospective validation.

## 4. Rational Combination Strategies for ADCs in BTC

While Antibody–Drug Conjugates (ADCs) are engineered to overcome the therapeutic limitations of naked mAbs by merging high-affinity targeting with potent cytotoxic payloads, they too have limitations. Despite their ability to enhance anti-tumor activity, ADCs still face significant pharmacological obstacles such as restricted parenchymal penetration within desmoplastic microenvironments and the emergence of resistance through antigen loss or compensatory signaling bypass. These persistent limitations suggest that even the best ADC monotherapy may be insufficient to eradicate complex and heterogeneous BTCs. Consequently, there is a clear clinical imperative to transition toward rational combination strategies. Synergistic partners are expected to deepen initial responses, delay the onset of acquired resistance, and convert the typically ‘cold’ BTC microenvironment into a more treatment-sensitive state [1]. By integrating these therapies, clinicians can address the multi-faceted nature of tumor evasion and maximize the durability of the therapeutic response.

### 4.1. Dual HER2 Blockade

A logically rational combination strategy is the established dual HER2 blockade consisting of trastuzumab plus pertuzumab. While this approach does not utilize an ADC, it serves as a critical clinical precedent by demonstrating that specific HER2 pathway inhibition can translate into meaningful activity in HER2-positive BTC. In the MyPathway phase 2a basket study, this combination achieved objective responses in a subset of heavily pretreated patients with manageable safety. This experience provided the framework for modern ADC-based strategies, specifically prompting the investigation of HER2-ADCs in combination with pertuzumab to further enhance therapeutic pressure. From these early successes, two fundamental lessons emerged for future ADC combinations. First, rigorous patient selection is essential, requiring validated HER2 positivity via immunohistochemistry (IHC) or in situ hybridization (ISH) alongside adequate tumor sampling. Second, the inherent variability of HER2 dependence and the challenge of intratumoral heterogeneity in BTC necessitate combination approaches to broaden and deepen the clinical benefit [68].

**Figure 2:**
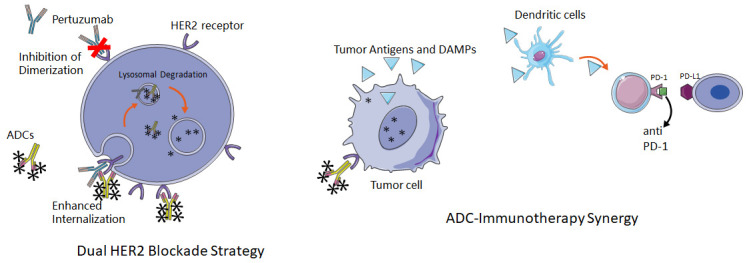
Schematic representation of rational combination strategies for ADCs in biliary tract cancer.

Following the success of dual monoclonal antibody blockade, the logical progression is to integrate an ADC to enhance tumor cell cytotoxicity while maintaining target specificity. The strongest proof of concept for this approach originates from HER2-positive breast cancer. In the DESTINY-Breast 09 trial, interim analysis presented at ASCO indicated that the combination of T-DXd plus pertuzumab improved progression-free survival compared with standard first-line therapy, achieving a high objective response rate. This result was reported as an interim analysis at ASCO [69]. While breast cancer is biologically distinct from BTC, its underlying design principle is highly informative, as pertuzumab may enhance HER2 pathway blockade and complement ADC payload delivery. In BTC, such a strategy is particularly relevant when HER2 signaling remains an important driver but intratumoral heterogeneity limits the durability of single-agent activity. This rationale is the foundation for the HER2-TRIP trial (NCT07129018), which explores the efficacy and safety of a novel quadruple regimen: SHR-A1811 (trastuzumab rezetecan) combined with pertuzumab and iparomlimab and tuvonralimab as a first-line treatment for HER2-expressing locally advanced or metastatic BTC [70]. By combining ADC-directed cytotoxicity with dual-pathway inhibition and immune modulation, this study seeks to optimize first-line treatment strategies and improve long-term survival rates (Figure 2).

(A) Dual HER2 Blockade Strategy: The combination of an HER2-directed ADC (e.g., T-DXd) with a dimerization inhibitor (e.g., pertuzumab) targets distinct epitopes on the HER2 receptor (e.g., Domain IV and Domain II, respectively). Pertuzumab inhibits ligand-independent HER2 heterodimerization (e.g., HER2-HER3), effectively blocking downstream oncogenic signaling. Crucially, the simultaneous binding of both agents promotes receptor clustering and cross-linking on the cell surface. This triggers enhanced receptor-mediated endocytosis (co-internalization) and subsequent lysosomal trafficking, thereby maximizing the intracellular concentration of the cytotoxic payload compared to ADC monotherapy.

(B) ADC-Immunotherapy Synergy: ADC payloads (such as topoisomerase I inhibitors) induce immunogenic cell death (ICD) in tumor cells, resulting in the release of tumor-associated antigens (TAAs) and damage-associated molecular patterns (DAMPs, such as calreticulin, ATP, and HMGB1). These danger signals recruit and mature dendritic cells (DCs), which subsequently prime and activate effector CD8+ T cells, potentially converting an immune-excluded (“cold”) tumor microenvironment into an inflamed (“hot”) one. The concurrent administration of immune checkpoint inhibitors (e.g., anti-PD-1/PD-L1 antibodies) blocks the inhibitory interaction between T cells and tumor cells, preventing T-cell exhaustion and amplifying the antitumor immune response initiated by the ADC.

### 4.2. ADC Plus Immunotherapy

A key rational partner for ADCs is immune checkpoint blockade. The goal is not only adding two effective drugs but also to use ADC-induced tumor cell death to increase antigen release and antigen presentation and then use PD-1/PD-L1 blockade to prevent adaptive immune resistance (Figure 2). Previous studies have shown T-DXd combined with an anti-PD-1 antibody produced better survival than either monotherapy, and the complete responses were more frequent. This type of evidence supports moving HER2-ADC plus checkpoint blockade into BTC where baseline immune suppression is common [30].

SHR-A1811-215 (NCT06778031) is an open-label, multicenter phase II study in HER2-positive locally advanced or metastatic BTC, designed for first-line combination treatment. The protocol uses a dose-exploration step for SHR-A1811, while the immune agents keep fixed doses. Two regimens are evaluated, including SHR-A1811 plus SHR-1316 (PD-L1 inhibitor), and a triple regimen adding SHR-8068 (CTLA-4 inhibitor). The primary endpoint is ORR, and key secondary endpoints include DCR, DoR, PFS, and OS [71].

### 4.3. The Ongoing Clinical Exploration

Current ADC combination trials in BTC are mainly focused on HER2 and CLDN18.2. For HER2, ongoing studies include T-DXd-based regimens (including the phase III DESTINY-BTC01 program) and HER2-ADCs such as disitamab vedotin and SHR-A1811 combined with PD-1/PD-L1 blockade (in some protocols with an added CTLA-4 component). For CLDN18.2, the main direction is a CLDN18.2-ADC combined with immunotherapy, such as a PD-1/CTLA-4 bispecific antibody. Beyond HER2 and CLDN18.2, an early-phase master protocol is evaluating the B7-H4-directed ADC AZD8205 with a dedicated BTC cohort and combination sub-studies (e.g., with the PARP inhibitor AZD5305 and immunotherapy modules), but BTC-specific outcomes have not yet been reported (NCT05123482) (Table 3) [40,71,72,73,74,75,76,77].

## 5. Challenges and Future Directions

In BTC, the main challenges for ADC development and clinical use are related to biology, safety, and patient selection. Tumor heterogeneity and variable antigen distribution mean that a positive result on a single biopsy does not always reflect the whole tumor, and the dense stromal background may further limit drug penetration, so efficacy can be inconsistent across patients. Safety is another key limitation, especially for DXd based ADCs, because interstitial lung disease (ILD) and pneumonitis can be serious and even fatal, as reported in BTC cohorts and in tumor-agnostic studies, so baseline lung assessment, early imaging review, and prompt steroid treatment are essential in routine practice. In BTC, liver function is also a practical barrier, since many patients have cholestasis or elevated bilirubin, and the prescribing information for T-DXd notes that dosing is not established in severe hepatic impairment and recommends close monitoring in moderate impairment, which can affect trial eligibility and real-world feasibility.

Future directions should keep exploring rational combinations, expanding the target landscape, and dynamic precision monitoring. Specifically, the next phase of clinical research in BTC must focus on refining the therapeutic index and establishing robust biomarkers. Clinical trials can be designed to include patients with moderate hepatic impairment, a demographic currently underrepresented but prevalent in real-world practice. Rationally designed “umbrella” trials could efficiently test multiple ADC-ICI combinations to define optimal sequencing. Additionally, as toxicities like ILD remain a barrier, future studies could incorporate digital health tools for early symptom monitoring. Ultimately, the field can move toward a comprehensive “precision ADC” model, where treatment choice is dictated not just by IHC positivity but by a composite score of antigen density, internalization rate, and payload sensitivity determined via early comprehensive molecular profiling. Harmonized assays, clear cutoffs, and dynamic monitoring, such as ctDNA-based reassessment, are important to make ADC treatment more reproducible. Despite the current paucity of high-level clinical evidence, the integration of molecular imaging represents an emerging area in precision oncology. By visualizing target accessibility and engagement in real time, these modalities may serve as critical tools for stratifying candidates for ADC therapy and evaluating treatment efficacy beyond standard anatomical criteria.

## 6. Conclusions

In summary, ADCs offer a uniquely suitable therapeutic concept for BTC because they combine target-selective delivery with highly potent payloads, and it can also generate a bystander effect that helps solve the intratumoral heterogeneity typical of BTC. Importantly, ADCs can also serve as an important component of combined therapy by inducing tumor cell death, which can enhance antigen release and immune activation, providing a biological basis for pairing with immune checkpoint blockade. In the meantime, combination programs in BTC are already expanding beyond HER2 to additional targets and immunotherapy partners. Collectively, as the ongoing trials refined the selection and combination strategies of the targets, the ADC-based approach holds great potential, offering deeper and more sustained responses and enabling the expansion of the effective treatment options for BTC.

## Figures and Tables

**Table 1:** Clinical landscape of ADCs in BTC.

Target	ADC	Payload	Linker	Biomarker	Study	Line of Therapy	Phase	Results Available	Trial
	Agent		Type	Definition	Type				Identifier
HER2	Trastuzumab deruxtecan	Cleavable (Tetrapeptide)	Cleavable	IHC 3+/2+	BTC-specific	Refractory/ Intolerant	II	Yes	NCCH1805 [32]
			(Tetrapeptide)						
	Trastuzumab deruxtecan	Topo-I inhibitor (DXd)	Cleavable	IHC 3+/2+	Pan-tumor with BTC cohort	Pre-treated	II	Yes	NCT04482309 [33]
			(Tetrapeptide)						
	Disitamab vedotin	Microtubule inhibitor (MMAE)	Cleavable	IHC 3+/2+	BTC-specific	Failed 1st-line Chemotherapy	II	No	NCT04329429 [34]
			(Val-Cit)						
	Zanidatamab zovodotin (ZW49)	Auristatin (ZD02044)	Cleavable	HER2	Pan-tumor including BTC cases	Advanced/Metastatic	I	No	NCT03821233 [35]
			(Protease-labile)	positive					
EGFR × HER3	BL-B01D1	Dual-ADC/cytotoxic payload	Cleavable	N/A	Pan-tumor with BTC cohort	Refractory/Intolerant	I	No	NCT05262491 [36]
			(Cathepsin B)					(Abstract)	
EGFR	MRG003	N/A	Cleavable	EGFR	BTC-specific	After ≥1 prior systemic therapy	II	No	NCT04838964 [37]
			(Val-Cit)	Positive					
TROP2	Sacituzumab govitecan	Topo-I inhibitor (SN-38)	Hydrolyzable (CL2A)	N/A	BTC-specific	Previously Treated	II	No	NCT06178588 [38]
	OBI-902	Topo-I inhibitor	Cleavable (GlycOBI^®^)	TROP2 positive	Pan-tumor including cholangiocarcinoma	Refractory/Intolerant	I/II	No	NCT07124117 [39]
	Datopotamab deruxtecan (Dato-DXd)	Topo-I inhibitor (DXd)	Cleavable (Tetrapeptide)	N/A	Pan-tumor with BTC cohort	Pre-treated	II	No	NCT05489211 [40]
CLDN18.2	LM-302 + cadonilimab	MMAE	Cleavable	CLDN18.2 Positive	BTC-specific	Failed standard therapy	I/II	No	NCT05994001 [41]
B7-H4	AZD8205 (puxitatug samrotecan)	Topo-I inhibitor	Cleavable	B7-H4	Pan-tumor with BTC cohort	Advanced/Refractory	I/IIa	No	NCT05123482 [42]
			(Peptide linker)	Positive				(Abstract)	
B7-H3	Ifinatamab deruxtecan (I-DXd)	Topo-I inhibitor (DXd)	Cleavable (Tetrapeptide)	N/A	Pan-tumor with BTC cohort	≥2nd line	1b/2	No	NCT06330064 [43]

Abbreviations: HER2, Human Epidermal growth factor Receptor 2; EGFR, Epidermal Growth Factor Receptor; Trophoblast cell surface antigen 2; CLDN18.2, Claudin 18 isoform 2; B7-H4, B7 Homolog 4.

**Table 2:** Emerging and exploratory ADC targets in BTC.

Target	ADC Agent	Payload	Development Status
B7-H3	Ifinatamab deruxtecan (I-DXd)	Topo-I inhibitor (DXd)	Phase Ib/II (NCT06330064)
ICAM1	ICAM1-ADCs	Topo-I inhibitor/MMAE	Preclinical
GPC1	Anti-GPC1-ADC	Auristatin (MMAF)	Preclinical
CD44v5	Anti-CD44v5-ADC	Auristatin (MMAE)	Preclinical
TA-MUC1	DS-3939a	Topo-I inhibitor (DXd)	Phase I/II (NCT05875168)

Abbreviations: CD44v5, CD44 variant 5; DXd, deruxtecan; GPC1, glypican-1; ICAM1, intercellular adhesion molecule 1; I-DXd, ifinatamab deruxtecan; MMAE, monomethyl auristatin E; MMAF, monomethyl auristatin F; TA-MUC1, tumor-associated mucin 1.

**Table 3:** Ongoing antibody–drug conjugate (ADC) combination trials in BTC.

Target	Strategies	ADC Agent	Payload	Combination Drug(s)	Line of Therapy	Phase	Recruitment Status	Registry ID
HER2	ADC + PD-1/PD-L1 ± dual-checkpoint	T-DXd ± rilvegostomig	Deruxtecan (DXd)	rilvegostomig; Gem/Cis + durvalumab	First-line	III	Recruiting/accepting sites listed	NCT06467357 [72]
	ADC + PD-L1	Disitamab vedotin (RC48)	MMAE	durvalumab	Advanced/	II	Pending/not yet recruiting	ChiCTR2200065807 [73]
					Metastatic			
	ADC + PD-L1	Disitamab vedotin (RC48)	MMAE	envafolimab	First-line	II	Not yet recruiting	NCT05417230 [74]
	ADC + PD-1	Disitamab vedotin (RC48)	MMAE	GLS-010/ zimberelimab	Previously Treated	II	Active/recruiting	NCT05540483 [75]
	ADC + PD-1/CTLA-4 bispecific	Disitamab vedotin	MMAE	cadonilimab	Advanced/	II	Recruiting	NCT06383533 [76]
					Metastatic			
	ADC + anti-angiogenic + PD-1	Disitamab vedotin	MMAE	lenvatinib + PD-1 inhibitor	Advanced/	II	Not yet recruiting	NCT07159217 [77]
					Metastatic			
	ADC + PD-L1)± CTLA-4	SHR-A1811	SHR169106	SHR-1316 ± SHR-8068	First-line	II	Recruiting	NCT06778031 [71]
B7-H4	ADC + PARP inhibitor ± immunotherapy	AZD8205	Topo-I	rilvegostomig	Advanced/	I/IIa	Recruiting	NCT05123482 [42]
			inhibitor		Refractory			
CLDN18.2	ADC + PD-1/CTLA-4 bispecific	LM-302	MMAE	cadonilimab	Post-Standard Therapy	I/II	Recruiting	NCT05994001 [41]

Abbreviations: ADC, antibody–drug conjugate; BTC, biliary tract cancer(s); HER2, human epidermal growth factor receptor 2; DXd, deruxtecan; MMAE, monomethyl auristatin E; PD-1, programmed cell death protein 1; PD-L1, programmed death-ligand 1; CTLA-4, cytotoxic T-lymphocyte-associated protein 4.

## Data Availability

No new data were created or analyzed in this study.
